# Importance of Reactive Agility and Change of Direction Speed in Differentiating Performance Levels in Junior Soccer Players: Reliability and Validity of Newly Developed Soccer-Specific Tests

**DOI:** 10.3389/fphys.2018.00506

**Published:** 2018-05-15

**Authors:** Haris Pojskic, Erik Åslin, Ante Krolo, Ivan Jukic, Ognjen Uljevic, Miodrag Spasic, Damir Sekulic

**Affiliations:** ^1^Department for Health Sciences, Faculty of Human Sciences, Mid Sweden University, Östersund, Sweden; ^2^The Swedish Winter Sports Research Centre, Mid Sweden University, Östersund, Sweden; ^3^Faculty of Kinesiology, University of Split, Split, Croatia; ^4^Faculty of Kinesiology, University of Zagreb, Zagreb, Croatia

**Keywords:** football, pre-planned agility, non-planned agility, conditioning capacities, team sports

## Abstract

Agility is a significant determinant of success in soccer; however, studies have rarely presented and evaluated soccer-specific tests of reactive agility (S_RAG) and non-reactive agility (change of direction speed – S_CODS) or their applicability in this sport. The aim of this study was to define the reliability and validity of newly developed tests of the S_RAG and S_CODS to discriminate between the performance levels of junior soccer players. The study consisted of 20 players who were involved at the highest national competitive rank (all males; age: 17.0 ± 0.9 years), divided into three playing positions (defenders, midfielders, and forwards) and two performance levels (U17 and U19). Variables included body mass (BM), body height, body fat percentage, 20-m sprint, squat jump, countermovement jump, reactive-strength-index, unilateral jump, 1RM-back-squat, S_CODS, and three protocols of S_RAG. The reliabilities of the S_RAG and S_CODS were appropriate to high (ICC: 0.70 to 0.92), with the strongest reliability evidenced for the S_CODS. The S_CODS and S_RAG shared 25–40% of the common variance. Playing positions significantly differed in BM (large effect-size differences [ES]; midfielders were lightest) and 1RM-back-squat (large ES; lowest results in midfielders). The performance levels significantly differed in age and experience in soccer; U19 achieved better results in the S_CODS (*t*-test: 3.61, *p* < 0.05, large ES) and two S_RAG protocols (*t*-test: 2.14 and 2.41, *p* < 0.05, moderate ES). Newly developed tests of soccer-specific agility are applicable to differentiate U17 and U19 players. Coaches who work with young soccer athletes should be informed that the development of soccer-specific CODS and RAG in this age is mostly dependent on training of the specific motor proficiency.

## Introduction

Agility has been defined as “a rapid whole-body movement with change of speed or direction in response to a stimulus” (Sheppard and Young, 2006). This definition is based on a model that separates agility into two components, the change of direction speed and perceptual and decision-making processes. The definition and model have been generally accepted for agility-based sports with several exceptions and additions (Brughelli et al., 2008; Chaouachi et al., 2012). Successful performance in team sports, such as soccer, requires change-of-direction ability, but also well-developed perceptual and decision-making skills that are evidenced by superior anticipatory motor performance (Bate, 1996; Gabbett et al., 2008; Gabbett and Benton, 2009; Lesinski et al., 2017; Loturco et al., 2017). Thus, it has been suggested that agility is one of the key performance indicators and therefore a fitness skill-related component that should be a part of standard physiological testing for soccer players (Svensson and Drust, 2005).

On the basis of a previously described idea of the existence of two capacities in agility, two independent types of agility performances are identified, including pre-planned agility (closed skill agility, change of direction speed – CODS) and non-planned agility (open skill agility, reactive agility – RAG) (Gabbett et al., 2008; Sekulic et al., 2014a; Spasic et al., 2015). The CODS enables athletes to outperform their opponents in situations in which they can pre-define the movement pattern (Sisic et al., 2016). In contrast, the RAG is accentuated when athletes perform a change in direction while reacting to an external stimulus (e.g., the trajectory of the ball, an opponent’s change in direction; Scanlan et al., 2015; Sekulic et al., 2017). However, the RAG and CODS are generally considered independent qualities. Studies to date have shown relatively low correlations between tests of these two capacities; thus, the independent assessment and development of these qualities are of substantial importance (Pehar et al., 2017b).

To date, several attempts have been made for RAG test development that is both valid and reliable. In the early phases of test development, authors introduced the “Y-shaped” RAG test that requires participants to sprint linearly and react accordingly with a change of direction without stopping after the visual signal (right or left; Sheppard et al., 2006; Serpell et al., 2010). Because their intention was to develop a rugby-specific test, these maneuvers were a logical experimental approach. In addition, similar RAG tests have been performed in soccer and involved only two reaction options (i.e., right or left) in a non-stop fashion (McGawley and Andersson, 2013; Di Mascio et al., 2015; Fiorilli et al., 2017). Moreover, as a result of sport-specific requirements, there is an evident trend of the development of sport-specific tests, including tests aimed to evaluate different types of agility performances.

For example, Spasic et al. (2015) presented the handball-specific test of reactive agility that consists of handball-specific stop-and-go movements, including forward running, lateral displacement, and backward running (Spasic et al., 2015). The presented test was shown to be a reliable and valid tool in the evaluation of handball-specific defensive performance (Spasic et al., 2015). In a more recent study, Sekulic et al. (2017) evaluated the reliability of a newly constructed basketball-specific reactive agility test and compared its discriminative validity with generic and basketball-specific CODS tests (Sekulic et al., 2017). The results showed appropriate reliability of all measurement tools assessed; however, the newly constructed reactive-agility test was the best predictor of player performance level. Moreover, in this study, the authors introduced the novel concept of testing while including a simple ball handling technique, which enabled them to mimic the real game reactive-agility performance that appears in basketball (Sekulic et al., 2017). Recently, Italian authors also recognized the importance of ball handling in soccer-specific agility performances, modified the original Y-shape test while adding the manipulation with the ball, and elegantly introduced the concept of the “technique-index” (i.e., the difference between agility performance with and without the ball). The main idea of the study was to evaluate this measurement approach for the identification of optimal field position (position in game) for young soccer players (Fiorilli et al., 2017). In general, their results did not enable the differentiation of soccer playing positions on the basis of measured and calculated variables. However, the experimental approach from this study highlighted the importance of sport-specific testing of agility in soccer, a concept that has also been recognized in other team sports (Spasic et al., 2015; Sekulic et al., 2017).

Previous studies indicate that apart from the eventual addition of a specific “ball-manipulation technique,” sport-specific tests of agility should take into account the specificity of the movement technique that appears in each sport of interest (Scanlan et al., 2015; Sisic et al., 2016). For example, soccer athletes more often than not have to change direction with various options repeatedly throughout “stop-and-go” movements. Thus, they often perform turns, alternate between running and lateral shuffling, and change from forward to backward running. The main difference between “stop-and-go” scenarios and the previously described “Y-shaped-course” scenario is that the latter lacks a moment of “zero velocity” (i.e., “Y-shaped-course” agility consists of non-stop running). The distinction between “non-stop” and “stop-and-go” agility has been proven in studies that demonstrated separate predictors for these 2 scenarios (Sekulic et al., 2013, 2014b; Spasic et al., 2013). Therefore, it appears reasonable to conclude that the “Y-shaped course” may not be an appropriate reactive agility test for all sports, including soccer (Serpell et al., 2010).

From previous literature overview, it is evident that CODS and RAG should be considered as vital components for successful performance in team sports including soccer (Bate, 1996; Sheppard and Young, 2006; Brughelli et al., 2008; Gabbett and Benton, 2009; Chaouachi et al., 2012; Spasic et al., 2015; Fiorilli et al., 2017; Sekulic et al., 2017). However, as a result of the absence of soccer-specific reactive agility tests that involve specific stop-and-go movement patterns and ball handling technique, the main rationale for this study was to determine whether newly developed tests of the RAG and CODS will be valid and reliable in the evaluation of soccer-specific agility performances. Therefore, the aim of this study was to evaluate the reliability and discriminative validity of newly designed testing protocols aimed at the evaluation of soccer-specific RAG and CODS in youth soccer players. More precisely, we assessed the discriminative validity of the newly developed measurement tools relative to tests of other conditioning capacities in differentiating players of two age categories (U17 and U19 teams). We hypothesized that the newly developed tests of soccer-specific CODS and RAG will depict the differences between the investigated age groups better than other tests of conditioning capacities (i.e., sprinting speed, jumping performances, and strength indices).

## Methods

### Study Design

**FIGURE 1:**
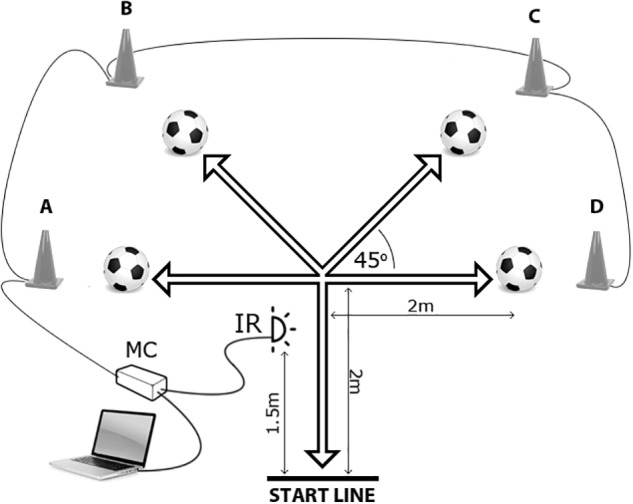
Testing of the soccer specific change of direction speed and reactive agility. MC, microcontroller; IR, infrared beam.

This cross-sectional field-based study consisted of three phases. In the first phase, we consulted with several experts (soccer coaches) from a club that participated at the highest level in Sweden regarding the agility movement patterns that are relatively common across all playing positions. In addition, they were instructed to determine the key situations in soccer that would be applicable for testing the agility performance of all athletes, regardless of their primary playing duties in soccer. These experts agreed that stopping the opponent’s first touch with the ball and pass interception with return to a starting position would be highly applicable for this purpose. In general, this defensive skill consists of a: (i) quick forward movement, (ii) diagonal/lateral movement, and (iii) a backward turn and run to a starting position (**Figure 1**). The second phase involved testing of all participants. In the third phase, the reliability and discriminative validity of the applied tests were established by determining the differences between the performance levels of soccer players (U17 vs. U19).

### Participants

Twenty young male soccer players [age: 17.0 ± 0.9 years; body height (BH): 1.81 ± 0.03 m; body mass (BM): 70.05 ± 7.41 kg], who participated at the highest level of competition in Sweden at their age, were recruited for this study. Participants were recruited if they were currently playing first grade soccer at their age group; had at least 6 years of experience in playing soccer; had a general soccer training history (more than three times per week) in the previous 6 months; were currently training for soccer (more than 8 h per week); and did not have existing medical conditions that would compromise study participation. For the purpose of defining the differences between performance levels, the players were divided into two performance level groups: younger (under 17 years of age; *N* = 10) and older (under 19 years of age; *N* = 10). In general, the time of the involvement in soccer was significantly different between the groups [11.8 ± 2.7 and 9.3 ± 1.8 years for the older and younger age groups, respectively (*p* < 0.05)]. Both groups had a similar training volume with a training frequency of 6–10 sessions per week. Athletes were in the preparation period and underwent approximately 5 weeks of regular soccer training before testing was conducted. Goalkeepers were excluded from the study. The ethics board of the first author’s institution provided the approval of the research experiment (Ethical Board Approval No: 2016/457-31). All participants were informed of the purpose, benefits, and risks of the investigation. The participants voluntarily participated in the testing after they provided written consent or after obtaining the parents’ written consent for participants younger than 18 years.

### Variables and Measurement

The testing procedures included measurements of BM, BH, body fat percentage (BF%), 20-m sprint (S20M), squat jump (SJ), countermovement jump (CMJ), drop jump (DJ), unilateral jump (UniJ), and back squat (SQUAT1RM), as well as the newly developed tests of soccer-specific CODS (S_CODS) and soccer-specific RAG (S_RAG). Testing occurred over 4 sessions: a familiarization session and 3 experimental sessions. The testing days were separated by 48 h of rest. To avoid diurnal variation, the testing sessions were performed between 10 and 12 am. The participants were provided with verbal encouragement and were instructed to use as much effort as possible during all tests. A standardized warm-up of approximately 10 min in duration was performed at the beginning of all testing days. This warm-up included a general warm-up, dynamic stretching and specific warm-up exercises. The general warm-up consisted of 800 m of running that was progressively increased in running speed at 90, 70, 60, and 45 s each 200 m (8 km/h, 10 km/h, 12 km/h, and 16 km/h). The dynamic stretching included front and lateral lunges, squats with dynamic exercise for the leg adductors, and exercises for the gluteus and gastrocnemius muscles. This was followed by a specific warm-up with high-intensity exercises: six vertical jumps (performed from <90° of the knee flexion angle) and two sub-maximal (70%) and maximal (95–100%) sprints. After the warm-up, there was an active rest of 3–5 min prior to the testing.

#### Familiarization Session

Prior to familiarization, the participants’ height and body mass were determined using a wall-mounted stadiometer and self-calibrating digital scale (MarQuant, Germany), respectively. The body fat percentage was subsequently calculated using the body density (BD) according to the following formula: BD = 1.162 2 0.063 3 log S4KN (where S4KN = the sum of the biceps, triceps, subscapular, and suprailiac skinfolds). The BD was converted to body fat percentage: BF% = (4.95/BD - 4.5) × 100 (Sisic et al., 2016).

The participants subsequently underwent three trials for each test in the study. Research personnel demonstrated the proper form for the execution of all tests. The participants were required to perform two to three trials to demonstrate technique proficiency and procedure familiarity. This was of substantial importance because of the intention to develop new RAG and CODS tests. The participants were instructed to perform agility tests as fast as possible and to identify the best movement strategies for themselves. Previous studies within the field have reported that familiarization is a crucial component as athletes typically find a preferable movement repertoire that enables them to achieve their best result (Sekulic et al., 2014b).

#### Testing Session 1

On the first testing day, S20M was used to measure the acceleration and speed qualities. The participants stood 1 m behind the start line in a middle stance starting position with the body leaned forward. Timing gates (Muscle Lab, Norway) were placed on the start (0 m) and finish (20 m) lines, with reflectors at 1 m in height. The participants were instructed to avoid backward movements before the start and sprint maximally throughout the whole distance without a “dive finish.” The athletes performed three attempts with a 2-min rest period between each sprint. The best score was used for the analyses.

#### Testing Session 2

During the second testing day, the maximal strength and explosive power were assessed using (SQUAT1RM), vertical jumps (CMJ), (SJ), (UniJ), and (DJ) to evaluate the athletes’ reactive strength index (RSI). Those tests were used because of their good reliability and high validity for assessing lower body maximal dynamic strength and explosive power (Markovic et al., 2004; McGuigan and Winchester, 2008). All jumps had instructional similarities. The participants were required to hold their hands on their hips during each attempt to avoid arm swing contribution. They were instructed to land on their toes at the same spot as their take-off. All jumps were performed three times with 1.5 min of rest between the attempts. The best score was used for the analysis. Jumps were assessed on a contact mat (Muscle Lab, Norway).

For the SQUAT1RM, the players were tested for a 6RM (six-repetition maximum) back squat, on a basis of the protocol suggested by Kraemer et al. (2006). The obtained number of repetitions (six) and a load were subsequently used to calculate the estimated 1RM using the equation by Brzycki (1993). To enable a better comparison between the players of different body masses, we normalized the 1RM squat results for body size using the allometric parameter *b* = 0.67 (Kukolj et al., 1999; Jaric et al., 2005).

For the SJ, the participants performed a maximal vertical jump from a starting position of 90–100 degrees of knee flexion. The athletes were required to jump as high as possible without countermovement before the jump. This was visually observed by an experienced examiner.

For the CMJ, the participants started from an upright position and were instructed to perform a downward movement that was immediately followed by a fast upward movement to enable the highest possible jump height (JH). The jump was used to test the stretch-shortening-cycle (SSC) utilization.

The UniJ was performed in the same fashion as the regular CMJ but with a single leg jump. The index of asymmetry (IA) was calculated as the difference (expressed as a percentage) in the JH between the legs.

When performing the DJ, the participants started in an upright position standing on a wooden drop box (25 cm). They were instructed to step forward of the box without stepping down or jumping up. At contact with the ground, they were instructed to shorten the contact time (CT) and maximize the JH as much as possible. The RSI was calculated as JH/CT (m/s) as previously reported, and the highest score was used for the analysis (Sattler et al., 2015).

#### Testing Session 3

The agility performances were tested with one protocol that evaluated the S_CODS and three protocols for the S_RAG, and the testing was performed on plastic turf grass. All performances were tested with the same equipment and test set-up, with the difference that the participants in the S_CODS protocol were aware of the movement pattern in advance. In contrast, the participants had no advanced knowledge of the testing scenario when they performed the S_RAG testing protocols. Each protocol consisted of five trials.

Measurements were performed using a hardware device system based on an ATMEL micro-controller (model AT89C51RE2; ATMEL Corp, San Jose, CA, United States) as the core of the system. A photoelectric infrared (IR) sensor (E18-D80NK) was used as an external time triggering input, and LEDs were used as controlled outputs. The photoelectric IR sensor has been shown to be as reliable as high-speed sensors, with a response time of less than 2 ms (500 Hz) and a digital output signal. The sensor’s detection distance ranged from 3 to 80 cm and was capable of detecting transparent or opaque objects. Because it has a digital output (high-low state) with an NPN transistor open collector, the sensor is connected through a microcontroller IO port. For the purposes of our study, this device was connected to a laptop PC operated on Windows 7. This equipment has previously been used and proven to be both valid and reliable for reactive agility and CODS assessments (Spasic et al., 2015; Sekulic et al., 2017).

The S_CODS and C_RAG were performed in the testing area shown in **Figure 1**. The participants commenced from the start line, and the timing was initiated when they crossed the IR signal. At this particular moment, a hardware module (microcontroller – MC) lit one of the four LEDs placed inside the 30-cm-high cones (labeled A, B, C, and D). When tested on the S_RAG, the participant had to assess which cone was lit, run to the particular cone, kick (rebound) the ball in front of the cone placed at the specially constructed stand positioned 3 cm above the ground, and return to the start line as quickly as possible. When a participant crossed the IR signal on their way back, the timing stopped. Testing of the S_RAG was performed over three protocols (S_RAG_P1_, S_RAG_P2_ and S_RAG_P3_), and the participants had no advanced knowledge of the testing scenario. The scenario for the S_RAG_P1_ included a light signal at the position A-C-B-D-C, with the S_RAG_P2_:B-C-A-D-A and the S_RAG_P3_: D-C-A-D-B. The participants performed the protocols in a random order. Following the reliability analysis (refer to the results on reliability), the best achievement for each of the three protocols was employed as the final result for each participant. The rest period between attempts was 10–15 s with 3 min of recovery between the protocols. The testing of the S_CODS was similar to the testing of the S_RAG performances; however, a participant had advanced knowledge of which cone would light up and only one protocol that consisted of five attempts was performed (scenario: A-B-C-D-A-). Following the reliability analysis, the best achievement was retained as the final result for each participant.

### Statistical Analyses

The reliability was established by calculating intra-class coefficients (ICC, model 3,1). Additionally, standard error of measurement (SEM; square root of mean-square-error derived from ANOVA for repeated measurements) and coefficient of variation (CV; [CV% = (SEM/Mean) × 100]) were calculated (Hopkins, 2000; Weir, 2005). The smallest worthwhile change (SWC) was computed as 0.3 of the between-subjects standard deviation (Hopkins, 2001). The Kolmogorov–Smirnov test identified all variables as normally distributed; therefore, descriptive statistics included the means and standard deviations. The homoscedasticity of the variables was tested by Levene’s test.

Pearson’s product–moment correlation was calculated to establish the associations between results obtained at S_CODS and three S_RAG protocols.

In order to identify differences among playing positions one-way ANOVA was calculated, with consecutive Scheffe *post hoc* analysis. To evaluate the effect sizes (ES) for differences among playing positions, partial eta squared values (η2) were presented (small ES: >0.02; medium ES: >0.13; large ES: >0.26; Cohen, 1988; Ferguson, 2009). The Student’s *t*-test for independent samples was used to evidence any possible difference in studied variables between playing levels (U17 vs. U19). Additionally, magnitude-based ES with 95 Confidence Intervals (CI) were calculated to establish differences between pairs of groups (i.e. defenders vs. midfielders, midfielders vs. forwards, defenders vs. forwards, and U17 vs. U19) using the following criteria: <0.02 = trivial, 0.2–0.6 = small, >0.6–1.2 = moderate, >1.2–2.0 = large, and >2.0 very large differences (Hopkins, 2000).

## Results

The reliability of the S_RAG and S_CODS is presented in **Table 1**. The ICCs ranged from appropriate to high values (0.70–0.92), with the strongest reliability evidenced for the S_CODS. Moreover, the highest CV was evidenced for the S_CODS (5.85%). The achievement for the S_CODS was 20–25% better than the achievement for the S_RAG protocols.

**Table 1:** Reliability and descriptive parameters for the soccer-specific change of direction speed test and reactive-agility tests.

	Mean ±*SD*	SWC	SEM	CI95% of SEM	CV%	ICC
S_CODS (s)	7.69 ± 0.45	0.14	0.13	7.44–7.94	5.85	0.92
S_CODS-1 _trial1_ (s)	7.82 ± 0.54				7.03	
S_CODS-2 _trial2_ (s)	7.66 ± 0.46				6.12	
S_CODS-3 _trial3_ (s)	7.59 ± 0.42				5.58	
S_RAG_P1_ (s)	9.9 ± 0.36	0.12	0.1	9.70–10.10	3.66	0.7
S_RAG_P1_trial1_ (s)	10.21 ± 0.57				5.59	
S_RAG_P1_trial2_ (s)	9.8 ± 0.37				3.87	
S_RAG_P1_trial3_ (s)	9.69 ± 0.4				4.19	
S_RAG_P2_ (s)	9.53 ± 0.44	0.13	0.12	9.29–9.77	4.71	0.88
S_RAG_P2_trial1_ (s)	10.16 ± 0.62				6.14	
S_RAG_P2_trial2_ (s)	9.81 ± 0.45				4.65	
S_RAG_P2_trial3_ (s)	9.55 ± 0.45				4.81	
S_RAG_P3_ (s)	9.99 ± 0.49	0.15	0.14	9.72–10.26	4.94	0.87
S_RAG_P2_trial1_ (s)	10.42 ± 0.63				6.09	
S_RAG_P2_trial2_ (s)	9.94 ± 0.53				5.36	
S_RAG_P2_trial3_ (s)	9.62 ± 0.47				4.99	


*S_CODS, the soccer-specific change of direction speed test; S_RAG, the soccer-specific reactive-agility test; _*P1*-*P3*_, three different protocols of S_RAG; SD, standard deviation; SWC, smallest worthwhile change; SEM, standard error of measurement; 95% CI, 95% confidence interval; CV%, coefficient of the variation; ICC, Intraclass correlation coefficient.*

The correlations between the S_CODS test and the three applied protocols of S_RAG evidenced that the S_CODS and S_RAG performances shared 25–40% of the common variance (*r* = 0.50, 0.56, and 0.63, for correlation between S-CODS with S_RAG_P1,_ S_RAG_P2,_ and S_RAG_P3_, respectively (all *p* < 0.05; **Table 2**).

**Table 2:** Pearson’s product moment correlations between soccer specific change of directions speed and reactive agility performances.

	S_RAG_P1_	S_RAG_P3_	S_RAG_P3_
S_CODS	0.50^∗^	0.56^∗^	0.63^∗^^∗^


*S_CODS, the soccer-specific change of direction speed test; S_RAG, the soccer-specific reactive-agility test; _*P1*-*P3*_, three different protocols of S_RAG; ^∗^ indicates statistical significance of *p* < 0.05; ^∗∗^ indicates statistical significance of *p* < 0.01.*

Playing positions significantly differed in body mass (large ES) and SQUAT1RM (large ES differences). For the remaining variables, no significant differences were identified among the three playing positions (small to medium ES obtained by η2; **Table 3**).

**Table 3:** ANOVA differences among three playing positions (defenders, midfielders and forwards) for the observed variables.

	Defenders	Midfielders	Forwards	ANOVA	
	Mean ±*SD*	Mean ±*SD*	Mean ±*SD*	*F*-test (*p*)	η^2^
Age (years)	17.00 ± 0.76	16.57 ± 0.98	17.6 ± 1.14	1.75 (0.21)	0.17
Years played (years)	11.00 ± 1.6	10.71 ± 3.04	9.6 ± 3.65	0.42 (0.66)	0.48
BH (m)	1.82 ± 0.04	1.81 ± 0.03	1.82 ± 0.03	0.14 (0.87)	0.02
BM (kg)	71.75 ± 5.57	62.71 ± 1.98^D^	77.60 ± 5.46^M^	16.16 (0.00)	0.65
BF (%)	11.47 ± 1.96	11.41 ± 2.72	10.88 ± 1.28	0.12 (0.88)	0.01
S20M (s)	2.99 ± 0.09	3.03 ± 0.1	3.05 ± 0.09	0.52 (0.60)	0.06
SJ (cm)	35.79 ± 2.42	34.0 ± 3.85	33.22 ± 4.24	0.97 (0.39)	0.1
CMJ (cm)	38.01 ± 2.6	36.59 ± 3.97	35.22 ± 3.11	1.15 (0.33)	0.12
IA (%)	11.82 ± 10.63	13.11 ± 5.59	5.4 ± 3.06	1.80 (0.20)	0.21
RSI (m/s)	1.55 ± 0.17	1.54 ± 0.22	1.4 ± 0.19	0.89 (0.43)	0.12
SQUAT1RM (kg)	104.9 ± 8.17	83.3 ± 8.21	104.7 ± 11.72^M^	9.82 (0.00)	0.62
R-SQUAT 1RM (kg)	1.44 ± 0.16	1.34 ± 0.14	1.36 ± 0.06	0.75 (0.49)	0.11
S_CODS (s)	7.59 ± 0.44	7.53 ± 0.42	7.35 ± 0.55	0.23 (0.79)	0.05
S_RAG _P1_ (s)	9.65 ± 0.44	9.46 ± 0.32	9.56 ± 0.44	1.86 (0.18)	0.04
S_RAG _P2_ (s)	9.65 ± 0.64	9.48 ± 0.25	9.42 ± 0.31	1.27 (0.30)	0.05
S_RAG _P3_ (s)	9.43 ± 0.4	9.69 ± 0.44	9.59 ± 0.57	0.53 (0.59)	0.07


*BH, body height; BM, body mass; BF, body fat; S20M, sprint 20 m; SJ, squat jump; CMJ, Countermovement Jump; IA, index of asymmetry (the difference in the unilateral CMJ height between the legs); RSI, reactive strength index; SQUAT1RM, 1 repetition maximum in back squat; oxR-SQUAT1RM, relative strength in back squat; S_CODS, the soccer-specific change of direction speed test; S_RAG, the soccer-specific reactive-agility test; _*P1*-*P3*_, three different protocols of S_RAG; η^*2*^, partial eta squared; ^*D*^ indicates values significantly different from those obtained by defenders at *p* < 0.05; ^*M*^ indicates values significantly different from those obtained by midfielders at *p* < 0.05.*

**FIGURE 2:**
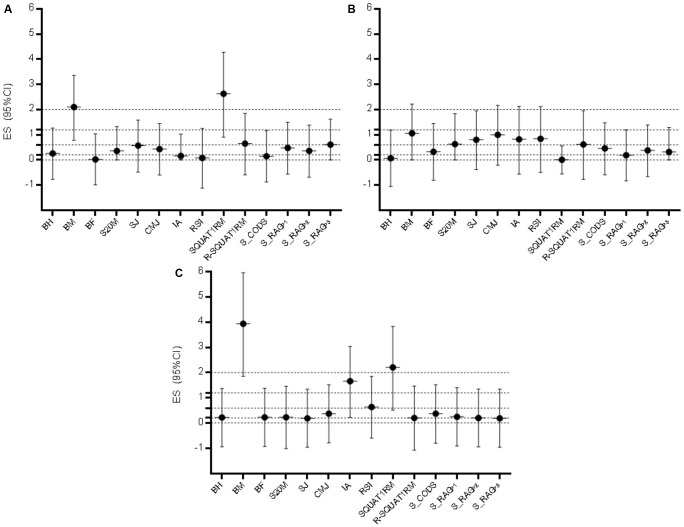
Effect size differences (Cohen’s d ES) for the observed variables with 95% Confidence Intervals (95%CI) between **(A)** defenders and midfielders, **(B)** midfielders and forwards, and **(C)** defenders and forwards. BH, body height; BM, body mass; BF, body fat; S20M, sprint 20 m; SJ, squat jump; CMJ, Countermovement Jump; IA, index of asymmetry (the difference in the unilateral CMJ height between the legs); RSI, reactive strength index; SQUAT1RM, 1 repetition maximum in back squat, R-SQUAT1RM, relative strength in back squat; S_CODS, the soccer-specific change of direction speed test; S_RAG, the soccer-specific reactive-agility test; P1–P3, three different protocols of S_RAG; dashed lines present ES ranges (<0.02 = trivial; 0.2–0.6 = small; >0.6–1.2 = moderate; >1.2–2.0 = large; and >2.0 very large differences).

**Figure 2** presents ES differences between playing positions based on Cohen’s d. When comparing defenders and midfielders, large ES were evidenced for BM, and very large for SQUAT1RM (defenders achieved higher results in both variables). The remaining ES differences between defenders and midfielders were trivial and small (**Figure 2A**). The ES differences between defenders and forwards were moderate for BM, S20M, SJ, CMJ, and IA (i.e., forwards were heavier, while defenders achieved better results in jumping performances and had higher IA; **Figure 2B**). The forwards were heavier (very large ES), and achieved higher result in SQUAT1RM (very large ES), in comparison to midfielders, while midfielders had higher IA (large ES), and RSI (moderate ES) than forwards, with small and trivial ES for remaining variables (**Figure 2C**).

The observed qualitative groups (U17 and U19) significantly differed in age (*t*-test: 2.62, *p* < 0.01; moderate ES), experience in soccer (*t*-test: 2.53, *p* = 0.03, moderate ES), S_CODS (*t*-test: 3.61, *p* < 0.05, large ES), S_RAG_P1_ (*t*-test: 2.14, *p* = 0.05, moderate ES), and S_RAG_P3_ (*t*-test: 2.41, *p* = 0.02, moderate ES). No significant differences between the two performance levels were identified for the observed anthropometric/body build indices or the remaining conditioning capacities (**Table 4**).

**Table 4:** Differences between the two performance levels (U17 and U19) for the observed anthropometric indices and the conditioning capacities (data are presented as Mean ± *SD*).

	U17	U19	*t*-test	ES (95%CI)
Age (years)	16.50 ± 0.7	17.5 ± 0.97	2.63**	1.17 (0.58-1.76)
Playing experience (years)	9.30 ± 1.88	11.8 ± 2.78	2.35*	1.05 (0.52-1.57)
BH (m)	1.82 ± 0.04	1.8 ± 0.02	0.80	0.36 (0.17-0.53)
BM (kg)	70.7 ± 9.14	69.4 ± 5.62	0.38	0.17 (0.08-0.25)
BF (%)	11.8 ± 2.05	10.8 ± 1.99	1.11	0.5 (0.25-0.74)
S20M (s)	3.03 ± 0.09	2.99 ± 0.08	0.78	0.37 (0.18-0.55)
SJ (cm)	33.3 ± 3.87	35.73 ± 2.59	1.64	0.77 (0.39-1.17)
CMJ (cm)	35.8 ± 3.75	37.8 ± 2.55	1.37	0.61 (0.30-0.91)
IA (%)	8.46 ± 5.88	12.13 ± 8.68	0.99	0.44 (0.22-0.66)
RSI (m/s)	1.47 ± 0.23	1.52 ± 0.16	0.46	0.23 (0.11-0.34)
SQUAT 1RM (kg)	92.9 ± 16	99.1 ± 11.9	0.85	0.43 (0.21-0.64)
R-SQUAT 1RM (kg)	1.32 ± 0.1	1.42 ± 0.13	1.57	0.81 (0.40-1.21)
S_CODS (s)	7.78 ± 0.33	7.22 ± 0.35	3.61***	1.61 (0.80-2.42)
S_RAG _P1_ (s)	9.73 ± 0.44	9.38 ± 0.24	2.14*	0.96 (0.48-1.43)
S_RAG _P2_ (s)	9.67 ± 0.42	9.38 ± 0.44	1.47	0.66 (0.32-0.98)
S_RAG _P3_ (s)	9.77 ± 0.44	9.34 ± 0.35	2.42*	1.1 (0.54-1.62)


*BH, body height; BM, body mass; BF, body fat; S20M, sprint 20m; SJ, squat jump; CMJ, Countermovement Jump; IA, index of asymmetry (the difference in the unilateral CMJ height between the legs); RSI, reactive strength index; SQUAT1RM, 1 repetition maximum in back squat; oxR-SQUAT1RM, relative strength in back squat; S_CODS, the soccer-specific change of direction speed test; S_RAG, the soccer-specific reactive-agility test; _*P1*-*P3*_, three different protocols of S_RAG; ES (95%CI), effect size with corresponding 95% confidence interval; ^∗^ indicates statistical significance of *p* < 0.05; ^∗∗^ indicates statistical significance of *p* < 0.01; ^∗∗∗^ indicates statistical significance of *p* < 0.001.*

## Discussion

Several important findings were obtained in this study. First, the newly developed tests aimed at the evaluation of soccer-specific CODS and reactive-agility were of appropriate reliability. Second, the playing positions did not substantially differ in the conditioning capacities assessed. Third, the newly developed tests of the S_CODS and S_RAG successfully discriminated U17 and U19 players, with better results in older players.

### Reliability

Previous studies have frequently reported the reliability of different types of agility tests that involve pre-planned (i.e., CODS) and non-planned (i.e., RAG) scenarios. In general, the tests varied in their reliability, with ICCs that ranged from 0.84 to 0.99 and 0.81 to 0.88 for the CODS and RAG tests, respectively. For example, strong reliability (ICC: 0.99) was identified for the modified version of the Illinois CODS test in a study that involved 95 youth soccer players (Hachana et al., 2014). Moreover, somewhat lower reliability (ICC: 0.87) was evidenced when academy rugby-union players were tested on sport-specific CODS, professional soccer players were tested on CODS via a non-stop movement template (ICC: 0.84), and college-level athletes were tested on the stop-and-go test of CODS (ICC: 0.81) (Mirkov et al., 2008; Green et al., 2011; Sekulic et al., 2014b). Therefore, we conclude that the S_CODS evaluated herein showed strong reliability (ICC: 0.92), especially bearing in mind that the S_CODS included (i) several changes of direction throughout a stop-and-go movement template and (ii) ball handling technique. Namely, it is established that the complexity of a test directly alters the consistency in the achieved testing results, and this is confirmed for tests of different conditioning capacities and various sports (Idrizovic et al., 2015; Pehar et al., 2017a;Sekulic et al., 2017).

The reliability of the S_RAG protocols varied (i.e., from ICC of 0.71 up to 0.88), However, it must be noted that the participants were randomly tested on the S_RAG protocols; therefore, differences among testing protocols with regard to reliability are likely a result of chance. The somewhat lower reliability of the S_RAG protocols (ICC: 0.70–0.88) than the S_CODS test (ICC: 0.92) is not surprising. Similar results are already noted in previous studies in which authors compared the reliability of the corresponding RAG and CODS procedures in college level athletes (Sekulic et al., 2014a), handball players (Spasic et al., 2015), and basketball players (Sekulic et al., 2017). In short, the RAG performance always includes perceptual and reactive components, which do not occur in the assessment of CODS. These additional “co-variates of performance” are naturally sources of mistake, potential sources of measurement error, and consequently factors that may alter the reliability. Therefore, the lower reliability of the S_RAG than the S_CODS may be attributed to the higher complexity of the reactive-agility tests as previously suggested (Sekulic et al., 2014a).

### Position Related Differences

Playing positions in soccer are well defined, and soccer players are involved in different tasks that are highly specific to different playing positions (Di Salvo et al., 2007). For example, defensive players must prevent opposing attackers from passing or receiving the ball and block their shots and passes. As a result, defenders are quick at anticipating opponents’ movements. Midfield players are often highly skilled and proficient in soccer ball handling technique. Because they connect defense and offense, they are excellent at ball control, have profound dribbling abilities, and are highly proficient in tactical knowledge. Finally, forwards must be capable of winning the ball (on the ground and in the air), receiving the ball, and scoring. Therefore, it is not surprising that studies have regularly identified body build and fitness-related differences between playing positions (Matkovic et al., 2003; Perroni et al., 2015).

It appears that the evolution of the soccer game has resulted in position-specific differences among players involved at different positions in a game. Namely, in older investigations, authors did not report anthropometric differences among defenders, midfielders, and forwards (Gil et al., 2007). However, it appears that the development of the sport has resulted in position specifics and consequently led to a position-specific fitness status of players. For example, Spanish authors in a more recent study reported significant anthropometric differences among playing positions similar to the results of our study (Lago-Penas et al., 2011). To some extent, the position specifics are also confirmed in our study. Namely, midfielders were lightest, which consequently resulted in the lowest results in absolute strength (SQUAT1RM); however, there was no significant difference among the groups in the relative strength (refer to **Table 3** and **Figure 2** for details).

As previously indicated, the only significant difference among playing positions is evidenced for the SQUAT1RM (i.e., midfielders achieved the lowest results). Although there were no differences in the relative strength, the difference in the SQUAT1RM should be attributed to the differences in body mass among the positions (i.e., midfielders are lightest of all positions). Of all investigated conditioning capacities, only the SQUAT1RM squat has been shown to be directly (positively) related to body mass (Stone et al., 2005). In support of this explanation, we highlight an almost identical effect-size among position differences for body mass and SQUAT1RM (η2: 0.65 and 0.62, respectively). One potential explanation for the small and non-significant differences in the conditioning capacities assessed among playing positions is that the studied players were not involved in position-specific conditioning programs to date. Consequently, they did not specifically develop the conditioning capacities related to their playing position duties (Di Salvo et al., 2007; Gil et al., 2007). Also, we must not ignore the fact that we grouped players into three playing positions only, whereas real-game playing positions in soccer are far more complex, which may influence our findings with respect to the non-significant differences in conditioning status among positions.

The importance of different facets of agility in team sports is well established (Di Salvo et al., 2007; Pehar et al., 2017b). Not surprisingly, studies have previously reported agility tests of appropriate validity in the differentiation of players involved in different playing duties (i.e., offensive vs. defensive players in handball; Spasic et al., 2015). Moreover, in a recent study conducted with youth soccer players, the authors initially aimed to verify whether the Y-shaped tests of CODS and RAG performed with and without the ball could be useful for the orientation of players toward playing positions in soccer (Fiorilli et al., 2017). No significant differences were identified among the different playing positions; as a result, the authors did not recommend the use of the applied tests as an indicator of appropriate playing positions in a soccer game. Therefore, our results regarding the non-significant position differences for the S_RAG and S_CODS support the recent findings of Fiorilli et al. (2017). However, as previously specified in the Section “Introduction” and Study design, our original intention was to design a measurement tool that will be applicable in the assessment of the reactive-agility of soccer players involved in different playing duties during a match. As the results indicated that there were no significant differences among playing positions in the S_CODS and S_RAG, we emphasize the developed measurement tools as being applicable in the assessment of different facets of agility among young soccer players irrespective of their playing positions.

### Performance Level Differences

Apart from tests of the S_CODS and S_RAG, U17 and U19 players did not differ in any other conditioning capacity investigated. It is even more intriguing given the significant difference in training experience between the groups (9.3 and 11.8 years in the younger and older groups, respectively). With regard to jumping performances, our results of the non-significant differences between performance levels are supportive of the majority of the studies within the field (Castagna and Castellini, 2013; Keiner et al., 2015). More specifically, Keiner et al. (2015) reported similar results of CMJ performance for German soccer players (33.7 ± 3.4 and 33.4 ± 3.4 cm for U17 and U19, respectively); moreover, Castagna and Castellini (2013) reported no significant difference for players involved in U17 and U20 Italian national teams (CMJ: 37.3 ± 4.7 and 38.0 ± 4.9 cm for U17 and U20, respectively). Furthermore, with regard to sprinting speed, Nikolaidis et al. (2016) and Slimani and Nikolaidis (2017) reported similar results for U17 and U19 players (S20: 3.16 and 3.09 seconds, respectively), which is supportive of our findings (i.e., no significant difference in S20 between performance levels). Meanwhile, some studies etc. performed on similar age groups have reported a superiority of older players in several conditioning capacities, with better jumping performance in older players. Specifically, Deprez et al. (2015) identified a significant difference between U17 and U19 in CMJ performance (34.3 ± 4.4 and 36.3 ± 4.3 cm, respectively), with better performance in older players . The finding that U19 participants achieved significantly better results than the U17 in the S_CODS and S_RAG is one of the most important findings of this study. First, this finding indirectly confirms that involvement in soccer training between the ages of 17 and 19 has a positive impact on the S_CODS and S_RAG, irrespective of other conditioning capacities (i.e., sprinting speed, reactive-strength, or jumping performance). Soccer-specific playing duties often challenge different facets of agility (Little and Williams, 2003). While CODS performances are challenged in situations in which a player is able to pre-plan the forthcoming movement template (mostly in offense), the reactive-agility is accentuated in situations in which a player must quickly and accurately react to external stimuli (i.e., movement of another player or ball trajectory). Taking into account that specific game duties in which the CODS and reactive-agility performances are challenged appear both in games and in training, the superiority of U19 in agility performances may be observed as a direct consequence of their longer involvement in systematic soccer training (experience of 9.3 and 11.9 years for U17 and U19, respectively) and higher performance level. Although we could not identify a study in which differences in agility were assessed between the age groups examined (i.e., U17 and U19), our results are in accordance with the findings of a study in which the authors compared older players (i.e., seniors vs. juniors; Mujika et al., 2009). Briefly, the authors reported sprinting, jumping, dribbling, endurance, and agility as potential determinants of the performance level and identified soccer-specific endurance and agility as the most important determinants of success.

The significant differences in the CODS and RAG between performance levels are particularly important in light of the previously discussed non-significant differences between performance levels in other conditioning capacities. More precisely, studies conducted to date have regularly confirmed the importance of sprinting and jumping capacities in different CODS performances, whereas the reactive-strength index (RSI) is reported as an important determinant of reactive-agility (Sekulic et al., 2013; Sattler et al., 2015). Moreover, it must be noted that U17 and U19 players did not differ in body dimensions (BH and BM) or BF%, which is also noted as an important determinant of CODS in reactive-agility in previous investigations (Sisic et al., 2016). This is in accordance with previous studies in the field of sport-specific agility in other sports (i.e., handball) in which authors identified an advancement in agility irrespective of the level of other conditioning capacities that contribute to agility (i.e., sprinting or jumping) (Spasic et al., 2015). Specifically, in sport-specific settings, proper movement technique may be a more important determinant of the CODS and RAG than conditioning capacities and body dimensions (Spasic et al., 2015).

### Limitations

The main limitation of this study originates from the cross-sectional design. Therefore, although we observed two performance level groups involved in equal sport settings, the established differences may not be explicitly attributed to the advanced training status of the older group; they may be a result of other non-controlled factors (i.e., initial selection of players in two generations). Moreover, we intended to develop and evaluate soccer-specific tests of stop-and-go CODS and reactive-agility, and both tests included a simple ball handling technique. However, in real game settings, soccer players often execute agile movements while dribbling the ball. Therefore, although the tests presented and evaluated have been established to exhibit good validity, further studies are required to investigate dribbling-specific soccer agile performances. In further development of the soccer specific testing protocols, evaluation of different testing protocols which will include performance in fatigued conditions will be particularly interesting.

## Conclusion

This study confirmed the high reliability of the newly developed soccer-specific tests of RAG and CODS in youth players; moreover, the results did not confirm positional differences for RAG and CODS performances. Therefore, the proposed tests may be used as reliable testing protocols to evaluate RAG and CODS irrespective of the position played in a soccer game. However, the CODS and RAG are identified as independent qualities in young soccer players. Therefore, to objectively evaluate both facets of agility, independent testing of these qualities is warranted. In doing so, special attention should focus on familiarization with different testing protocols. This approach will enable each player to individually determine the most appropriate way to execute the test (s) and assure consistency of the collected test results.

The tests of S_RAG and S_CODS are applicable in the differentiation of the two performance levels. The U19 players achieved significantly better results than their younger and less-experienced peers (i.e., U17). Although U19 players achieved better results in agility performances, the jumping, sprinting, and reactive-strength performances observed in this study were similar across performance levels. As all players observed in this study were recruited from the same sport settings, these results may be at least partially attributed to the specific development of conditioning qualities in the period between 17 and 19 years of age.

The coaches and professionals who work with young soccer athletes should be aware that the development of the soccer-specific CODS and RAG during this age (i.e., between 17 and 19 years) is mostly dependent on the training of specific motor proficiency. At the same time, they must be informed that there is no evidence that the development of other capacities that are regularly considered important determinants of agility (i.e., sprinting, jumping, and reactive-strength) will have a positive impact on the development of soccer-specific agile performances for this age.

## Data Availability

The datasets for this study can be found in https://www.dropbox.com/s/uozjh7aelsdzsjz/Reactive-agility-in-soccer-data-2017.xlsx?dl=0.

## Author Contributions

HP and EÅ collected the data, performed statistical analyses, and participated in drafting the manuscript. OU, AK, and MS developed the testing equipment of the evaluation of the agility components and helped in data interpretation. IJ and DS gave an overview of the previous research and discussed the data. All authors participated in study design and substantially participated in final manuscript versions, approved the submitted version, and agreed to be accountable for all aspects of the work.

## Conflict of Interest Statement

The authors declare that the research was conducted in the absence of any commercial or financial relationships that could be construed as a potential conflict of interest.
